# Ewing Sarcoma Meets Epigenetics, Immunology and Nanomedicine: Moving Forward into Novel Therapeutic Strategies

**DOI:** 10.3390/cancers14215473

**Published:** 2022-11-07

**Authors:** Sara Sánchez-Molina, Elisabet Figuerola-Bou, Víctor Sánchez-Margalet, Luis de la Cruz-Merino, Jaume Mora, Enrique de Álava Casado, Daniel José García-Domínguez, Lourdes Hontecillas-Prieto

**Affiliations:** 1 Developmental Tumor Biology Laboratory, Institut de Recerca Sant Joan de Déu, Hospital Sant Joan de Déu, Esplugues de Llobregat, 08950 Barcelona, Spain; 2 Pediatric Cancer Center Barcelona, Hospital Sant Joan de Déu, Esplugues de Llobregat, 08950 Barcelona, Spain; 3 Clinical Laboratory, Department of Medical Biochemistry and Molecular Biology, School of Medicine, Virgen Macarena University Hospital, University of Seville, 41009 Seville, Spain; 4 Oncology Service, Department of Medicines, School of Medicine, Virgen Macarena University Hospital, University of Seville, 41009 Seville, Spain; 5 Institute of Biomedicine of Seville (IBiS), Hospital Universitario Virgen del Rocío/CSIC/University of Seville/CIBERONC, 41013 Seville, Spain; 6 Pathology Unit, Hospital Universitario Virgen del Rocío/CSIC/University of Seville/CIBERONC, 41013 Seville, Spain; 7 Department of Normal and Pathological Cytology and Histology, School of Medicine, University of Seville, 41009 Seville, Spain

**Keywords:** Ewing Sarcoma, epigenetic, immunotherapy, nanotherapy

## Abstract

**Simple Summary:**

Ewing Sarcoma treatment is traditionally based on chemotherapy, surgery, and radiotherapy. Although these standard of care regimens are efficient at early disease stages, many patients fail to respond appropriately, which has prompted the search for more efficacious and specific treatments. A deeper understanding of the basic molecular mechanisms underlying the biology of both tumor cells and the tumor microenvironment, as well as advances in drug delivery, has led to the development of different approaches to improve the treatment in Ewing Sarcoma patients. Thus, epigenetic, and immunotherapy-based drugs, along with nanotechnology delivery strategies, represent novel preclinical and clinical studies in the treatment of Ewing Sarcoma. In this review, we provide a comprehensive overview of these emerging therapeutic strategies and summarize the potential of the latest preclinical and clinical trials in Ewing Sarcoma research. Finally, we underline the value and future directions of these new treatments.

**Abstract:**

Ewing Sarcoma (EWS) is an aggressive bone and soft tissue tumor that mainly affects children, adolescents, and young adults. The standard therapy, including chemotherapy, surgery, and radiotherapy, has substantially improved the survival of EWS patients with localized disease. Unfortunately, this multimodal treatment remains elusive in clinics for those patients with recurrent or metastatic disease who have an unfavorable prognosis. Consistently, there is an urgent need to find new strategies for patients that fail to respond to standard therapies. In this regard, in the last decade, treatments targeting epigenetic dependencies in tumor cells and the immune system have emerged into the clinical scenario. Additionally, recent advances in nanomedicine provide novel delivery drug systems, which may address challenges such as side effects and toxicity. Therefore, therapeutic strategies stemming from epigenetics, immunology, and nanomedicine yield promising alternatives for treating these patients. In this review, we highlight the most relevant EWS preclinical and clinical studies in epigenetics, immunotherapy, and nanotherapy conducted in the last five years.

## 1. Introduction

Ewing Sarcoma (EWS) is a rare and highly aggressive bone and soft tissue tumor that affects children, adolescents, and young adults with a peak of incidence in the second decade of life. The prognosis of EWS has improved considerably, with current multimodal therapy including chemotherapy, surgery, and radiation, with a 65–70% cure rate for localized disease. However, older patients (>18 years), metastatic patients at diagnosis, and patients with early relapsing tumors still have a poor prognosis, with a 5-year survival rate of less than 30% [1,2]. Therefore, the higher therapeutic challenge remains on how to control the systemic disease and improve the survival rates, especially in those patients with worse prognosis.

EWS tumor cells are characterized by a fusion gene involving one member of the *FET* family of genes and one of the *ETS* family of transcription factors, *EWSR1-FLI1* being the most common [1,3]. Fusion genes have been demonstrated to be essential for tumorigenesis and, therefore, are attractive therapeutic targets that can be addressed through direct and indirect molecular targeted approaches [4]. Nevertheless, the lack of specific enzymatic activity of EWSR1-FLI1 challenges a direct targeted pharmacological inhibition. Moreover, indirect inhibition of oncogene activity by the perturbation of downstream targets, although it has presented successful integration in preclinical models, remains elusive in clinics [5].

Advances in the molecular mechanisms underlying the epigenetic remodeling of chromatin mediated by the fusion oncogene and the immune system have led to the development of novel therapeutic approaches. Epigenetic changes driven by EWSR1-FLI1 have been reported in the tumorigenesis of EWS. Indeed, EWSR1-FLI1 rewires chromatin and reprograms gene expression causing both induction and repression of selected gene pathways [6,7,8]. Therefore, epigenetic-based treatments provide a prominent option for treating this aggressive tumor by reversing the effect in the epigenome induced by the fusion gene. Moreover, based on the experience gained from adult cancer, immunotherapy studies have been translated to pediatric tumors including EWS.

There is a pressing requirement to develop targeted therapies or drug carriers that can deliver therapeutic agents with higher efficiency to lower the dosage needed and minimize side effects. On this basis, nanotechnology plays a prominent role in modern medicine, by potentially overcoming the deficiencies of conventional methods of administering chemotherapy and ultimately improving clinical outcomes [9].

In this article, we will revise the ongoing preclinical and clinical studies of the last five years focusing on epigenetics, immunotherapy and nanotherapy in EWS.

## 2. Epigenetic and Immunotherapy-Based Treatments in EWS: Moving Forward in Targeted Therapies

The ultimate knowledge of the basic aspects of the epigenetics and immunotherapy of cancer has made significant strides, leading to the development of a wide variety of new therapeutic agents. Here, we summarize the newest epigenetic and immune-based treatments in EWS.

### 2.1. Epigenetic Therapy

Epigenetics encompasses the reversible molecular processes affecting chromatin that define cellular identity by maintaining on and off states of transcription without alterations in the DNA sequence. Upon sequencing studies, different groups reported EWS as a tumor with paucity in the mutational rate, implicating epigenetics behind EWSR1-FLI1 as a tumorigenic factor [10]. As a result, many publications have shed light on the role of the EWS epigenome both in the understanding of the molecular mechanisms involved in tumor development and in the identification of novel targets for new and combinational therapies [11].

Epigenetics is critical to induce the proper environment for EWSR1-FLI1 establishment, as cells with higher plasticity will provide more significant opportunities for reprogramming by the oncogene [12,13]. Besides, the oncogene that interacts directly with DNA presents scaffolding properties that mediate protein–protein interactions with important epigenetic regulators of chromatin structure, rewiring the complete epigenome and, ultimately, their expression programs [14]. EWSR1-FLI1 behaves as a pioneer factor by directly recruiting chromatin remodelers to GGAA microsatellites, where it induces the formation of de novo active super-enhancers in regions that were previously repressed [11]. Finally, the repressive role of the oncogene is described by its capability to displace endogenous transcription factors [7]. Understanding the epigenetic mechanisms that permit cancer cells to quickly adapt, and their reversibility, therefore, constitutes a great opportunity for the development of new strategies to treat cancer [15]. The following sections will focus on those epigenetic drugs that can be translated into the clinics, which include targeting DNA methylation, nucleosome remodelers, histone post-translational modifications and their modifiers (Table 1).

#### 2.1.1. DNA Methylation

DNA methylation at cytosine (5-methylcytosine, 5mC) is an essential process in embryonic development and cell differentiation [16]. Disruption of the DNA methylation pattern is a common trait of different cancers, including EWS, where hypermethylation of key genes correlates with more aggressive behavior and hypomethylation was reported in active enhancers [17,18]. DNA methyltransferases (DNMT) and ten-eleven translocation (TET) methylcytosine dioxygenases, responsible for DNA demethylation, have been major targets for epigenetic drug development. Despite their high efficiency, DNMT inhibitors (DNMTi), such as azacitidine and decitabine, presented toxicity in phase I clinical trials and low doses in combination with other agents were further tested [19]. Recently, the novel non-nucleoside DNMTi MC3343 has been described to induce a specific depletion of DNMT1 that induces DNA damage without alterations in DNA methylation [20].

Besides, non-epigenetic drugs were reported to affect TET enzymes and histone demethylases. Mutations that disrupt isocitrate dehydrogenase IDH1/2 enzymatic function produce a reduction in α-ketoglutarate (αKG) and an increase in the oncometabolite 2-hydroxyglutarate (2HG). In particular, 2HG inhibits TET enzymes resulting in DNA hypermethylation; thus, drugs inhibiting mutant IDH1/2 reactivate αKG and restore methylation levels [6]. On this basis, ivodesinib, an inhibitor of mutated IDH1, is actually in phase II clinical trial for refractory and recurrent pediatric solid tumors including EWS (NCT04195555).

#### 2.1.2. Nucleosome Remodeling

Nucleosome remodeling refers to the ATP-dependent multiprotein complexes that affect nucleosome positioning and structure, influencing transcription regulation. Among these complexes, EWSR1-FLI1 recruits the nucleosome remodeling and deacetylase (NuRD) complex. This complex contains histone deacetylases (HDAC), lysine specific demethylase 1 (LSD1) and chromodomain-helicase-DNA-binding protein 3/4 (CHD3/4) and directly binds to EWSR1-FLI1 promoting transcriptional repression in EWS [21]. The inhibition of LSD1 with the non-competitive reversible LSD1 inhibitors HCI-2509 and HCI-2528 was effective in targeting EWS cell lines, while their efficiency was dependent on *EWSR1-FLI1* expression [21]. HCI-2509 delayed tumor growth in monotherapy [22] and its efficiency was not altered by the previous inhibition of EWS cell lines with the irreversible inhibitor GSK-LSD1, suggesting that HCI-2509 disrupts the LSD1 interaction with EWSR1-FLI1 [23]. Nevertheless, the latest studies have reported LSD1 colocalization at EWSR1-FLI1 active super-enhancers, correlating with HCI-2509 disruption not only of repression but also gene activation [24]. SP-2577 (seclidemstat), another LSD1 inhibitor, inhibited the growth of three out of eight EWS xenograft models [25]. At present, there are four clinical trials: (i) a phase I evaluating the safety-dose escalation and expansion of seclidemstat with topotecan and cyclophosphamide in patients with relapsed or refractory EWS (NCT03600649); (ii) a phase I/II as a continuation of a previous one, which allows the patient continued access to the drug (NCT05266196); (iii) a phase I study evaluating the safety and preliminary antitumor activity of INCB059872, another selective and oral LDS1 inhibitor, in refractory or relapsed EWS patients (NCT03514407); and (iv) a dose-escalation and dose-expansion study of INCB059872 in advanced solid malignancies including EWS (NCT02712905).

Among a panel of pediatric sarcoma cell lines, EWS cells were the most sensitive to trabectedin, an antitumor drug derived from the sea squirt that binds to the minor groove of DNA, reversing the gene signature of EWSR1-FLI1 by interference with its transcription factor activity [26]. EWSR1-FLI1 can also recruit the mammalian switch/sucrose non-fermenting (SWI/SNF) nucleosome remodeler to enhancers containing GGAA microsatellites facilitating chromatin opening and activation of EWSR1-FLI1-targets [27]. Later studies demonstrated that trabectedin evicted the SWI/SNF complex from chromatin and redistributed EWSR1-FLI1 within the nucleus, disrupting its function as a pioneer factor [28]. Although the phase I clinical trial in children with refractory solid tumors concluded that trabectedin was safe, a phase II study was unsuccessful [29,30]. A new phase II clinical trial combines trabectedin with radiation in advanced and metastatic EWS (NCT05131386), and another three evaluate the combination of trabectedin or its derivative lurbinectedin with irinotecan based on their synergy (NCT04067115, NCT05042934, NCT02611024 and [31]).

#### 2.1.3. Histone Modifications and Modifiers

Histone tails undergo a variety of post-translational covalent modifications that affect their interaction with DNA. The different histone modifications constitute a code where synergistic or antagonistic interactions determine chromatin accessibility to transcription factors and ultimately transcription activation or repression [32]. The enzymatic activities behind this histone code involve writers that settle these modifications (including histone acetyltransferases (HAT) or histone methyltransferases (HMT)), erasers, which eliminate them (including histone demethylases (HDM) or HDAC), and finally, readers that recognize and mediate an epigenetic signal.

##### Histone Writers: Polycomb Group and G9a Methyltransferase

The polycomb group (PcG) proteins segregate in two transcriptional repressive complexes, PRC1 and PRC2. PRC1 contains the E3 ubiquitin ligase enzyme RING1A or RING1B, while PRC2 consists of HMT activity from EZH1 or EZH2. Despite the repressive role of PRC1, RING1B has been described to be a transcriptional activator in various cancer entities [33,34]. In EWS, RING1B is highly expressed and is necessary for the expression of critical EWSR1-FLI1 targets by facilitating oncogene recruitment to active enhancers. Inhibition of aurora kinase (AURK) B by AZD1152 has been proposed as an excellent strategy to impair RING1B activity at active enhancers [35]. Moreover, EWS cells were highly sensitive to both AURKA and B inhibitors and their combination with focal adhesion kinase (FAK) inhibitors reduced the tumor growth in EWS mouse models [36].

The PRC2 subunit EZH2 is overexpressed in EWS and its knockdown inhibited tumor growth and metastasis in vivo [37,38]. Consequently, different EZH2 inhibitors have been evaluated in EWS in order to target PCR2 activity, such as the non-specific inhibitor 3-deazaneplanocin A (DZNep) and the specific inhibitor tazemetostat. DZNep treatment produced a cell cycle arrest in vitro and growth suppression in EWS mice [39]. The tolerability of tazemetostat is being evaluated in a phase II clinical trial in pediatric patients with gain of function mutations of EZH2 including EWS (NCT03213665). Nevertheless, tazemetostat showed no activity in four xenograft models of EWS [40]. Besides, EZH2 inhibitors combined with immunotherapy might offer a new therapeutic opportunity. It has been observed that GSK126, another selective EZH2 inhibitor, as well as tazemetostat, enhance the surface expression of disialoganglioside (GD2) in EWS cell lines, which sensitizes EWS cells to cytolysis by GD2-specific chimeric antigen receptor (CAR) T-cell immune therapy (see next chapter) [41].

Finally, G9a, an HMT that dimethylates H3K9, has been found to be overexpressed in different cancer types. Specifically, its overexpression in EWS correlated with poor prognosis and metastasis [42]. Indeed, the G9a inhibition with BIX01294 was proved effective in disrupting migration, invasion, adhesion, colony formation, and vasculogenic mimicry via the upregulation of *NEU1*. Decrease in metastasis and tumor growth with BIX01294 was proven in two in vivo models of EWS metastasis [42].

##### Histone Erasers: Deacetylases and Demethylases

HDAC antagonize the enzymatic activity of HAT by removing histone acetylation. EWSR1-FLI1 was shown to globally repress HAT activity while stimulating HDAC [43]. Consistently, several HDAC inhibitors (HDACi) were screened in EWS, including FK228 (romidepsin) and MS-275 (entinostat), which presented antitumor activity in vitro and in vivo in EWS, as well as vorinostat (SAHA) and sodium butyrate (NaB) [43,44,45]. Lessnick et al., showed that both vorinostat and depletion of HDAC2/3 reversed expression patterns of EWSR1-FLI1-repressed targets, indicating that the oncogene relies on HDAC for its repressive role in transcription [21]. Nevertheless, the first initial preclinical testing of vorinostat retrieved no objective responses for any of the solid tumors tested, including EWS [46]. Besides, entinostat, a selective HDAC1 and HDAC3 inhibitor, significantly reduced tumor burden and increased survival in preclinical xenograft models inducing cell cycle arrest and apoptosis. However, only the knockdown of HDAC3 was critical for EWS survival [47].

Further studies have revealed the potential of HDACi in combination with other drugs. A screening of 43 epigenetic drugs revealed that the most sensitive agents in EWS cell lines were related to HDAC inhibition, being BML-281, a specific inhibitor of HDAC6, the drug with the lower IC50. BML-281 increased acetylation levels of specificity protein 1 (SP1), reducing its binding to the *EWSR1-FLI1* promoter and causing repression of the oncogene and its associated targets [48]. Furthermore, the combination of the HDAC6 inhibitor ACY-1215 with doxorubicin reduced tumor growth in EWS xenografts [48]. On the other hand, *HDAC1* and *HDAC2* knockouts demonstrated a reduction in invasiveness and tumor growth in xenografts [49]. Since the effect in tumor growth resembled EZH2 inhibition [37], the HDACi romidepsin was combined with the embryonic ectoderm development (EED) inhibitor (A-395), which inactivates the PRC2 complex. This combination treatment was superior to monotherapy blocking the proliferation and tumor growth of SK-N-MC or EW7 xenograft models [49]. In addition, the combination of SAHA with HCI-2509 decreased cell proliferation, triggering cell cycle arrest and apoptosis, reducing *EWSR1-FLI1* expression by regulation of the *EWSR1* promoter and altering tumor growth [50]. Along the same line, the combination of romidepsin with HCI-2509 has also proved to be synergistic [51]. Currently, a phase I clinical trial combining vorinostat with chemotherapy in refractory or relapsed solid tumors is open (NCT04308330). Interestingly, HDACi could be chemically modified to have a second pharmacophore, like fimepinostat, which is a hybrid inhibitor of phosphatidylinositol 3-kinase (PI3K) and HDACs. This drug not only reduced EWSR1-FLI1 protein by affecting its stability but also cell viability and tumor growth in sarcoma xenograft models [52].

Regarding histone demethylation, the Jumonji-domain HDM KDM3B demethylates H3K9me2, and has been described as a novel oncogene downstream of EWSR1-FLI1 [53]. KDM3B and its direct target, the cell adhesion molecule MCAM, were positively implicated in cell migration and invasion, and their knockout reduced metastasis in vivo [54]. Indeed, EWS cell lines were sensitive to the pan-selective Jumonji HDM inhibitor JIB-04, which increased methylation levels of H3K4me3, H3K9me2, and H3K27me3 and affected the whole EWSR1-FLI1 transcriptome. JIB-04 induced DNA damage via CDKN1A and decreased tumor growth in xenograft models [55]. Besides, a drug screening revealed that EWS cell lines were sensitive to the H3K27me3 demethylase inhibitor GSK-J4. This drug sensitized EWS cell lines to chemotherapy and synergized in vivo with the cyclin-dependent kinase (CDK) 7/12/13 inhibitor THZ1 [56]. Nevertheless, these new epigenetic drugs have not yet reached into the clinics.

##### Histone Readers: Bromodomains

The bromodomain and extra-terminal (BET) family consists of four conserved mammalian members (BRD containing 2 (BRD2), BRD3, BRD4, and BRDT) that interact through bromodomains with acetylated lysine residues [57,58]. The first BET inhibitor described was JQ1, a molecule that competitively binds to bromodomains, preventing the interaction between BET proteins and acetylated histones. In EWS, both JQ1 and depleted BRD proteins suppressed the *EWSR1-FLI1* gene signature. Besides, JQ1 compromised cell proliferation, angiogenesis, and tumor growth in EWS xenograft models [59,60]. BMS-986158 and BMS-986378, another two BET inhibitors, have now entered clinical trials as investigational drugs for evaluating their efficacy for pediatric brain and solid tumors (NCT03936465).

### 2.2. Immunotherapy

**Figure 1:**
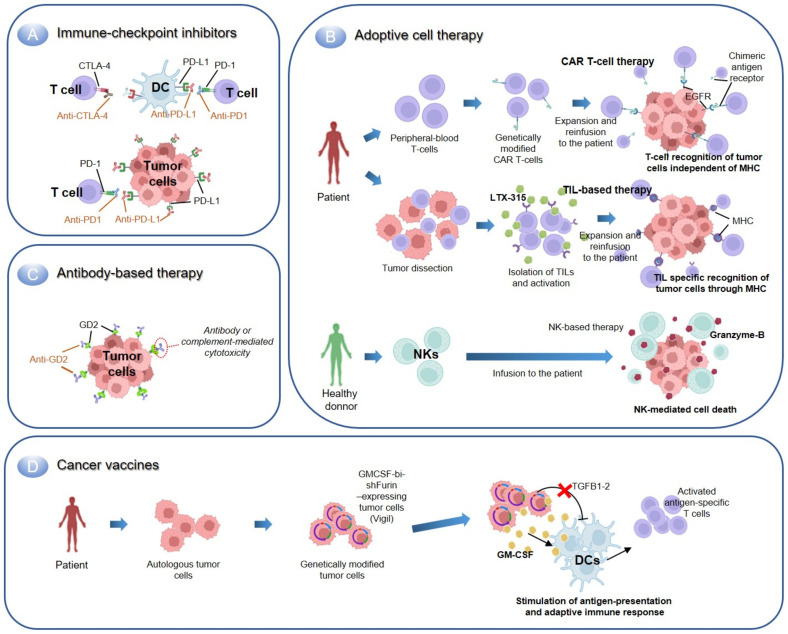
Immune therapies currently explored in EWS. (**A**) Immune checkpoint inhibitors block the interaction of immune checkpoint molecules (e.g., PD-1 or CTLA-4) with its inhibitory ligands to stimulate the immune response. (**B**) Adoptive cell therapy involves the infusion of modified autologous T-cells or allogenic NK cells. T-cells can be genetically modified to express a chimeric antigen receptor (CAR) specific of a tumor-associated antigen (e.g., EGFR) that can be recognized by major histocompatibility complex (MHC)-independent mechanisms. In contrast, T-cells isolated from tumors can be stimulated with an oncolytic peptide (e.g., LTX-315) and reinfusioned back to mediate an antitumoral MHC-dependent response. Transfer of NK cells from healthy donors is based on the innate ability of NK cells to kill tumor cells through various mechanisms such as granzyme B release. (**C**) Antibody-based therapies involve the use of specific antibodies targeting tumor-associated antigens (e.g., GD2). (**D**) Cancer vaccines stimulate the immune system response of the host through various mechanisms. The VIGIL vaccine in EWS is based on the tumor cells engineered to express GM-CSF and a bifunctional shRNA that prevents immunosuppression by TGFβ1-2 release. Reinfusion of these tumor cells, thus, promotes antigen-presentation and the adaptive immune response.

Immunotherapy is a treatment that boosts the immune system response against cancer or blocks any mechanism that prevents antitumor immunity. The local tumor microenvironment (TME) and the host immune system define the tumor immunophenotype, which is generally divided into hot and cold tumors. Whereas hot tumors resemble an immune-inflamed phenotype characterized by infiltration of T lymphocytes, cold tumors present an immune-desert or immune-excluded phenotype with the absence or exclusion of T-cells [61]. EWS exemplifies an immune cold tumor with very poor infiltration of immune cells or inflammatory infiltrates due to immune escape, immune privilege, or immune inhibition by the TME. Tumor cells resemble a deficient expression of human leukocyte antigens (HLA) that prevents recognition of tumor-associated antigens by effector T-cells and antigen presenting cells. Consistently, self or tumor-reactive T-cells extracted from EWS patients show an exhausted phenotype that failed to activate despite the presence of high doses of antigen [62]. In the same lines, immune-inhibitory ligands, such as HLA-G were found locally expressed on tumor cells and on infiltrating lymphocytes, which promote direct inhibition of the immune response by natural killer (NK) cells as well as the induction and expansion of myeloid-derived suppressor cells (MDSCs) [63,64]. Besides, large populations of MDSCs were shown to inhibit EWS immune responses to therapy [65]. While a better understanding of the interplay between EWS and TME is being developed, novel immunotherapy strategies are focused on increasing the number of T-cells driving them into the tumor and reversing the immunosuppressive TME [66]. These therapies include immune checkpoint inhibitors, adoptive cell therapy, antibody-based immunotherapy, and cancer vaccines, which are addressed below (Figure 1).

#### 2.2.1. Immune Checkpoint Inhibitors

Immune checkpoint molecules are inhibitory and stimulatory ligand–receptor pairs that exert an inhibitory or stimulatory effect on immune responses. They are usually expressed in T-cells to maintain self-tolerance and regulate the magnitude of the immune response. Additionally, these molecules have been described as participating in immune evasion in cancer [67]. Blocking the interaction of checkpoint molecules by immune checkpoint inhibitors (ICI) is currently under research to increase T-cell activation and proliferation, causing T-cell cytotoxicity towards tumor cells. ICI treatment typically targets PD1 or CTLA4 immune checkpoint molecules, which have shown promising clinical efficacy in various solid tumors, including melanoma [68,69]. Three trials have studied the efficacy of ICI in pediatric sarcomas showing no benefit for EWS patients. In a phase I trial, ipilimumab (anti-CTLA4) was evaluated in children and adolescents with sarcoma, however, it showed no remarkable benefit considering the small sample size [70]. Next, a multicentric study evaluated pembrolizumab (anti-PD1) in advanced sarcomas, reporting an objective response in only 18 and 5% of soft tissue and bone sarcoma, respectively, although no response in the 13 EWS patients was observed [71]. The last trial studied the combination of both anti-PD1 and anti-CTLA4 and confirmed the limited efficacy of anti-PD1 in monotherapy, while reporting modest benefits of the combination in some sarcoma subtypes beyond EWS (5% and 16% overall response rate, respectively) [72]. The tumor mutation burden contributes to the immune recognition of cancer cells and, together with the expression of both PD-1 and PD-L1, seem to predict the response to ICI treatment [73,74]. On this basis, the low mutation rates of EWS and the fact that these tumors have a low expression of PD1 or its ligands (25.7% and 19.2%, respectively) might explain the poor response of these tumors to ICI. Moreover, another study reported PD-L1 expression in 33% of EWS, which significantly anticorrelated with survival [75,76].

New therapeutic strategies beyond ICI focus on combining these agents. VEGF promotes an immunosuppressive microenvironment and contributes to ICI resistance in cancer [77]. Consistently, clinical trials are combining pembrolizumab with VEGFR inhibitors (NCT02636725, NCT05182164). The combination of pembrolizumab with the VEGFR inhibitor axitinib has shown low toxicity and preliminary activity in a phase II trial, although no remarkable response was reported for EWS patients [78] (NCT02636725). Another phase II study is assessing the efficacy of combining pembrolizumab with cabozantinib, a receptor tyrosine kinase inhibitor, in patients with advanced sarcomas (NCT05182164). Additionally, a phase I/II trial with sequential administration of nivolumab (anti-PD1) and escalating doses of the mTOR-inhibitor ABI-009 has been conducted with EWS patients in which the efficacy and safety of the treatment will be evaluated (NCT03190174). The last results of this study showed no dose-limiting toxicities [79]. NKTR-214 is an engineered version of the interleukin 2 receptor (IL-2R) with a polyethylene glycol chain (bempegaldesleukin or BEMPEG) that reduces IL-2 binding to CD25 over CD122. Consequently, a sustained activation of antitumor CD8^+^ T-cells and NK cells, which is associated with tumor regression, is promoted [80]. Novel studies indicate the benefit of combining this therapy with ICI [81]. On this basis, a non-randomized two part open-label trial is evaluating the safety, tolerability, and dose level of the combinatory treatment of nivolumab with BEMPEG, as well as the efficacy of the combination in children and young adults with recurrent or refractory tumors including EWS (NCT04730349). However, trials with this combination have been discontinued recently.

Finally, B7 homolog 3 (B7-H3) is a checkpoint inhibitory protein of the B7-CD28 family that is overexpressed in multiple cancer types including osteosarcoma, whose expression is associated with poor survival [82]. Enoblituzumab (MGA271) is a humanized IgG1 monoclonal antibody targeting B7-H3 that is being trialed in children with relapsed or refractory malignant solid tumors with high expression of B7-H3, including osteosarcoma, EWS, neuroblastoma, rhabdomyosarcoma, Wilms Tumor and desmoplastic small round cell tumors (NCT02982941). This phase I trial will determine its safety, tolerability, immunogenicity, and preliminary antitumor activity in these tumor entities.

#### 2.2.2. Adoptive Cell Therapy

In contrast to ICI therapy, which is intended to reinvigorate a suppressed or poor immune response against tumor, adoptive cell therapy (ACT) or cellular immunotherapy evades T-cell activation steps. On this basis, ACT involves the infusion of tumor-resident or peripheral blood-modified immune cells to promote an antitumor response, which includes the transfer of modified T-cells and NK cells.

##### Transfer of T-Cells

Tumor-infiltrating lymphocytes (TIL) are T-cells found in malignant tissues whose function and localization are critical to eventual tumor control or progression [83]. Consistent with the immune cold phenotype of EWS, a poor number of TILs are closely associated with deficient HLA expression in tumor cells that protects against immune recognition. Moreover, low expression of HLA-I is associated with poor survival in EWS patients [75,84]. Consequently, ACT therapies are seeking reinvigorating strategies, such as the infiltration of pre-stimulated TILs or genetically modified T-cells, for the patient. TILs’ collection and expansion from tumors is feasible, and reinfusion has shown cytotoxic responses against tumor [85]. Nevertheless, the pre-treatment conditioning of T-cells is important to enhance engraftment and persistence of transferred cell populations. This strategy is currently being explored in phase I/II clinical trials with advanced and metastatic sarcomas, in which TILs’ reinfusion to the patient is co-administered with or without a high dose of IL-2 (NCT04052334, NCT03449108). A phase II trial investigated the treatment of TILs with an oncolytic peptide (LTX-315), resulting in a feasible and tolerable combination with manageable toxicity in various metastatic sarcomas [86]. Other strategies focus on the infusion of T cells with a genetically modified T cell receptor (TCR) recognizing HLA-I restricted antigens uniquely expressed by tumor cells, which permits to identify intracellular antigens. The first clinical use of TCR transgenic T cells recognizing EWS-derived peptides (allorestricted) in EWS patients was directed against chondromodulin-1 (CHM1), a transmembrane glycoprotein directly activated by EWSR1-FLI1 that promotes metastatic spread [87]. Transfer of the HLA-A*02:01/CHM1^319^ TCR transgenic CD8^+^ T cells to three refractory EWS patients was well tolerated and was associated with disease regression, although this has not gone into clinical trials yet [88]. Furthermore, transferred T cells home into the affected bone marrow and persist, which gives hope to those patients with bone marrow metastasis that do not survive irrespective of therapy [89]. Other TCR-based therapies targeting the tumor-restricted expression of cancer testis antigens like NY-ESO-1 has been extensively studied in the context of sarcomas, with promising clinical results in synovial sarcoma [90,91]. Two phase I clinical trials with NY-ESO-1-based TCR therapies are currently ongoing in bone and soft tissue sarcomas (NCT03462316 and NCT03240861).

On the other hand, CAR therapies are based on the engineering of T-cells expressing a novel receptor designed to combine the effector properties of T-cells and the ability of antibodies to recognize pre-defined surface antigens of cancer cells with a high degree of specificity [92]. CAR-based therapies have been highly efficient for hematologic malignancies and around 470 clinical trials are now running [93,94]. However, multiple facts constraint its success in solid tumors, which includes T-cell limited survival and expansion, activation-induced cell death, T-cell exhaustion, trogocytosis, antigen loss, and unintended gene transduction of tumor cells [95,96]. Furthermore, designing CAR therapies is challenging in heterogeneous tumors such as EWS, where minimal “universally” membrane-expressed targets exist. GD2, aforementioned, has a 40–90% expression in primary EWS and thus has been used as a CAR-based target [97,98]. GD2-specific CAR T-cells were highly effective in patients with high-risk neuroblastoma [99], although no antitumor effect against GD2-positive EWS xenograft models was reported. However, investigators found MDSCs inhibited human CAR T cell responses in sarcomas and treatment with retinoic acid reduced the immunosuppressive capacity of MDSCs. These results suggested that retinoids enhanced the clinical efficacy of CAR therapies in sarcomas [65]. Novel therapies in tumors expressing high GD2, including EWS, explore the clinical effect of a GD2-CAR therapy in combination with chemotherapy with or without a previous lymphodepletion regimen (NCT03373097 and NCT03635632, respectively). Moreover, the combinatory effect of CAR-T-cells (targeting multiple markers like GD2), with low dose chemotherapy followed by maintenance with sarcoma vaccines is in a phase I/II trial (NCT04433221). Further approaches have designed CARs against the ICI molecule B7-H3, which has shown potent antitumor activity in EWS xenograft models [100]. Consistently, B7-H3-based CARs are now in phase I clinical trials in pediatric solid tumors including EWS (NCT04897321, NCT04483778). Finally, the epithelial growth factor receptor (EGFR) is another target for CAR therapy in EWS and its inhibition has an antitumor activity in vitro [101]. A phase I trial using EGFR-CAR (EGFR806) is recruiting relapsed patients with preliminary data indicating acceptable toxicity and antitumor activity in children and young adults (NCT03618381) [102].

Challenging clinical aspects of CAR therapies is the high toxicity reported, partially explained by the expression of CAR-targeting antigens in healthy tissues. The design of new generation CARs might overcome this issue. In this regard, larger phase I/II clinical trials are being conducted to study the safety and efficacy of 4th generation CAR T-cell therapies in various tumors, including EWS (NCT03356782).

##### Transfer of Natural Killer Cells

NK cells were named for their ability to kill cancer cells autonomously without antigen presentation. These cells express numerous inhibitory, activating, adhesion, and cytokine receptors that permit the direct recognition of cell-stress signals or foreign antigens to self-activate or suppress its cytolytic activity [103]. Considering the lack of neoepitopes in pediatric tumors, the innate ability of NK cells to recognize activating ligands on tumors is beneficial. A preclinical study showed that chemoresistant sarcoma cell lines, including EWS, were sensitive to NK cell killing in vitro and in vivo [104]. Moreover, investigators showed that EWS cells and primary tumors were susceptible to NK cytotoxicity through the expression of ligands for the activating NK cell receptors NKG2D and DNAM-1 and the use of cytokines increased the effectivity [105]. Additionally, transduced NK cells with a GD2-specific CAR has shown to enhance their ability to lyse cells in EWS in vitro [106]. On this basis, a phase I clinical trial explores this antitumor strategy by transplanting allogenic (donated) and previously stimulated NK cells in pediatric patients with solid tumors or leukemia (NCT01287104). NK cells usually are infused from a histocompatible donor. A phase I trial including EWS patients proposes using NK cells from unmatched healthy donors stimulated with the interleukin 15 agonist ALT-803, an experimental procedure that has not yet been approved by FDA (NCT02890758). Finally, results from a phase II clinical trial with the infusion of autologous NK cells in combination with sirolimus (mTOR inhibitor) maintenance strategy in relapsed patients have shown good tolerance with 45% 2-year overall survival (OS) and 25% of progression-free survival (PFS) in EWS patients [107]. The technical improvements of the last years in the expansion of NK cells ex vivo as well as the development of new platforms (like CARs or bispecific NK-cell engagers) that increase target specificity of NK cells makes this a promising immunotherapy strategy not only in sarcomas but in other pediatric tumors [108,109].

#### 2.2.3. Antibody-Based Immunotherapy

Treatment based on the usage of monoclonal antibodies (mAbs) emerged at least 30 years ago and are standard-of-care treatment nowadays for malignancies like breast cancer [110]. These therapies are based on the specific binding of mAbs targeting tumor-specific antigens, including the TME, which produces the killing of tumor cells through various mechanisms, as reviewed by Weiner [111]. Many studies aimed to use mAbs-based therapies in EWS clinical trials, as summarized in Table 2. For instance, mAbs targeting the insulin growth factor 1 (IGF-1) pathway have been explored extensively. The IGF-1 pathway is pivotal in EWS pathogenesis with studies showing that inhibition of IGF-1R reduced cell migration and tumor growth in vitro and in vivo [112,113,114,115]. However, clinical trials with anti-IGF-1 have shown an overall response rate of only 10–14% and a median PFS of less than 2 years [116,117,118]. Moreover, a randomized phase III clinical trial (NCT02306161) evaluated the use of ganitumab (targeting IGF-1R) with interval-compressed conventional chemotherapy in metastatic EWS patients, but this study was closed due to increased toxicities and lack of clinical benefit [119]. Further trials with ganitumab include its combination with palbociclib (NCT04129151), although lack of clinical benefit was reported [120]. Other mAbs targeting the IGF pathway have been analyzed in preclinical and clinical studies with relatively low response rates, as reviewed by Casey et al. [121]. Apart from IGF-1, mAbs targeting the VEGF pathway alone or in combination with chemotherapy have also been explored in sarcomas like EWS. Consistently, a randomized phase II clinical trial evaluated whether the addition of bevacizumab (targeting VEGF-R) to vincristine, cyclophosphamide and topototecan chemotherapy regimens could improve survival. However, the benefit to add bevacizumab was unclear [122]. Recent studies with the anti-murine DC101 targeting VEGF-R2 further support the rationale to target this pathway in EWS. They showed the administration of DC101 caused a delay in tumor growth of sarcoma PDX like EWS and its addition to chemotherapy resulted in an improvement of the anti-tumoral response [123]. A phase I clinical trial with the humanized version of DC101, ramucirumab, has been conducted in a range of pediatric patients with recurrent or refractory solid tumors, whose results are still missing (NCT02564198). Olaratumab (IMC-3G3) exemplifies another mAb-based therapy clinically explored in EWS, which targets the plateled-derived growth factor receptor (PDGFR). A phase I and randomized phase II study in patients with unresectable or metastatic soft tissue sarcoma reported to improve OS nearly 12 months when received olaratumab with doxorubicin compared to doxorubicin alone (NCT01185964) [124]. However, this was not confirmed in the following phase III trial (NCT02451943, ANNOUNCE) [125]. Subsequent trials evaluating the second-line addition of olaratumab to gemcitabine and docetaxel in advanced soft tissue sarcomas indicated no statistical significant improvement in the OS between the two arms (NCT02659020). However, the combination resulted favorable in the PFS and an objective response in both cohorts [126]. Additional studies have reported olaratumab combined with pembrolizumab is safe and well tolerated in patients with advanced soft tissue sarcomas, although further studies with an increased sample size are needed to evaluate the efficacy of these regimens (NCT03126591) [127].

Dinutuximab is a humanized GD2-mAb that was proved to benefit the survival of high-risk neuroblastoma patients and is now used for maintenance therapy. Hu14.18K322A is a derivative of dinutuximab developed to reduce allogeneic reactions. NCT02159443 trial will evaluate the presence of pretreatment anti-therapeutic antibodies that might influence Hu14.18K322A response in EWS and other malignancies.

In the last decades, mAb technology has improved by conjugating antibodies with various antitumor effector molecules, including cytotoxic drugs (named antibody-drug conjugates or ADC) and radioconjugates (RIC). ADCs are comprised of a mAb bound to a cytotoxic drug that selectively binds to target cells and directly delivers the toxic payload [130]. Endoglin (CD105) is a coreceptor of the TGFβ family associated with poor prognosis in EWS [131]. Targeting endoglin with a first-in-class ADC conjugated to a tubulin polymerization inhibitor showed potent preclinical activity in EWS, although this has not been explored clinically [132]. AXL has a high expression in EWS and was associated with worse OS. Moreover, its inhibition chemosensitized EWS cell lines to vincristine or doxorubicin [133]. Now a phase II clinical trial with EWS patients is exploring usage of AXL-mAb (BA3011) conjugated with monomethyl auristatin E (MMAE), a chemotherapy agent that blocks tubulin polymerization (NCT03425279). On the other hand, RIC is a combination of mAbs to radioisotopes that can be used for both radioimaging of tumor cells and pharmacologically targeting tumor cells [134]. Endosialin (TEM-1) is a cell surface glycoprotein expressed in advanced sarcomas that promotes tumor growth through the platelet-derived growth factor (PDGF) signaling [135]. Ontuxizumab (MORAb-004), a mAb that targets endosialin and blocks PDGF signaling, has been studied in phase I/II trials in EWS; however, no objective response was observed [129,136]. Recently, the endosialin-RIC [^111^In] CHX-DTPA-scFv78-Fc (with indium-111) has been evaluated preclinically in EWS cell lines showing potential to translate into clinics [137]. Finally, two phase I/II clinical studies with GD2 or B7-H3- targeting RIC are explored in sarcoma patients with dissemination in the central nervous system or leptomeningeal space (NCT00445965, NCT00089245). The B7-H3 study, including 9 pediatric sarcoma patients, has shown that intraventricular administration of the RIC mAb-therapy was safe and had a favorable dosimetry in the central nervous system, suggesting this might have clinical utility in patients with this type of dissemination [128].

#### 2.2.4. Cancer Vaccines

Cancer vaccines stimulate the immune system typically by recognizing tumor-associated antigens and include peptide or dendritic cell vaccines loaded with tumor lysate or pulsed with antigenic peptides. On this basis, a vaccine made of patient-derived dendritic immune cells loaded with autologous tumor lysate or tumor antigens ex vivo has been shown to activate an antitumor response, although poor response in phase I/II studies in EWS and other soft tissue sarcomas [138]. New efforts are focused on combining these vaccines with chemotherapeutic regimens. Vigil (formally known as FANG) is an autologous cancer cell vaccine that is engineered to express granulocyte-macrophage colony-stimulating factor (GM-CSF), which stimulates antigen-presentation in combination with a bifunctional shRNA-furin that prevents cleavage of TGF-β and reduces its local immunosuppressive effect. A prospective non-randomized study of advanced EWS patients reported 1-year survival of 73% for Vigil-treated patients compared to 23% in the control group historically treated with conventional therapy [139]. Given the very low toxicity reported, a randomized phase III clinical trial combining temozolomide and irinotecan with or without Vigil in relapsed EWS patients is currently under investigation, representing one of the few phase III trials for these patients (NCT03495921).

## 3. Nanotherapy: A Refined Target-Specific Drug Delivery System

Nanomedicine is a novel therapeutic strategy based on the application of nanotechnology to medicine through the development and use of nanoparticles (NPS). NPS have nanoscale dimensions (ranging from 1 to 100 nm in diameter) [140] with specific nanomaterial properties, which include surface charge, size, morphology, and area that compromise their activities and effects [141]. Considering all these variables, NPS can be classified based on (i) structure (flat, spherical, crystalline, etc.); (ii) dimensionality (one-, two- and three-dimensional NPS) [142]; (iii) porosity (porous and non-porous materials) [143]; and, (iv) chemical composition (organic, inorganic, carbon-based nanomaterials and hybrid nanostructures [142].

Among the fundamental advantages of nanomedicine usage is the improvement of diagnostic sensitivity, imaging, and radiation therapy, as well as a more precise and efficacy delivery of pharmaceutical agents to the targeted tissue [144,145]. Therefore, its main application in cancer is the delivery of chemotherapeutic drugs reducing side effects to the minimum and getting the maximum clinical benefit. For this reason, the number of preclinical and clinical studies has considerably increased in recent years.

### 3.1. Ewing Sarcoma Nano-Systems

**Figure 2:**
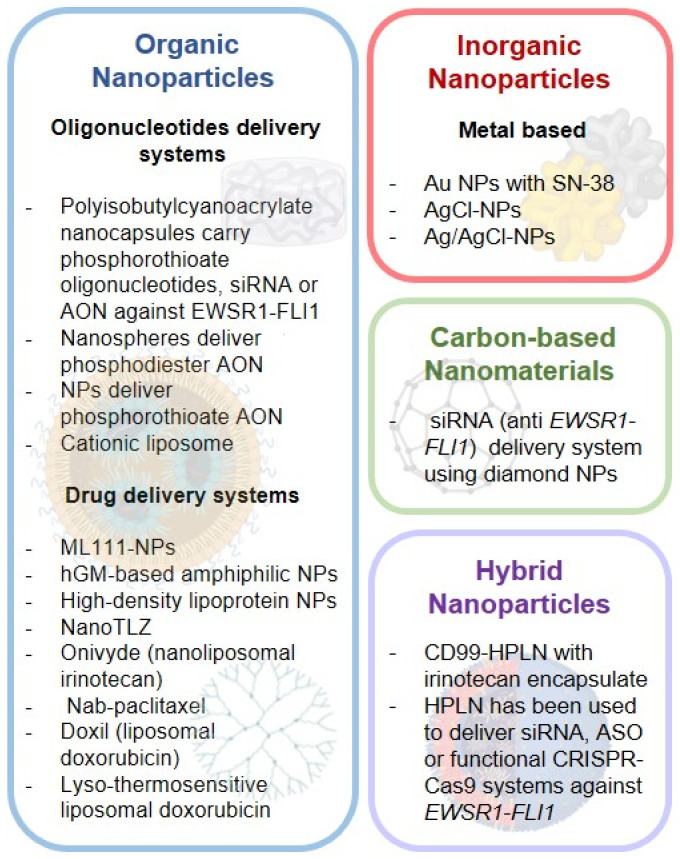
Summarizing the Nanoparticles and nanomaterials used in EWS studies.

The side effects associated with the administration of chemotherapy drugs and the innate and/or acquired chemotherapy resistance in EWS cells remain challenging. On this basis, drug delivery systems involving NPS refine some of these aspects, although the number of studies is still limited. The following sections describe novel preclinical and clinical studies in EWS based on the chemical composition of NPS (Figure 2 and Table 3).

#### 3.1.1. Organic NPS

Organic NPS, also named polymers, are the most widely used NPS in biomedicine, including cancer, due to their biodegradable and non-toxic properties. They include micelles, dendrimers, liposomes, hydrogels, among others [146]. EWS studies carried out to date with organic NPS can be divided into two groups: oligonucleotide and drug delivery systems.

##### Oligonucleotide Delivery Systems

On this basis, polyisobutylcyanoacrylate nanocapsules have demonstrated their ability to inhibit *EWSR1-FLI1*. These NPS allow to carry phosphorothioate oligonucleotides [147] or small interfering RNAs (siRNAs) against *EWSR1-FLI1* [148], which inhibited tumor growth on EWS mice xenografts, and *EWSR1-FLI1* expression [147,148]. Furthermore, phosphorotionate NPS and phosphodiester nanospheres carrying antisense oligonucleotides (AON) against *EWSR1-FLI1* were also used, showing both a reduction of EWS tumor growth in vivo when injected intratumorally [149]. Rao et al., have developed a bifunctional shRNA (bi-shRNA) target sequence against the *EWSR1-FLI1* fusion gene that was complexed with a cationic liposome (PBI-shRNA *EWSR1-FLI1* LPX), resulting in 85–92% of *EWSR1-FLI1* knockdown (protein and RNA) in vitro. PBI-shRNA *EWSR1-FLI1* LPX was used in EWS xenograft mice, confirming its efficacy and safety. However, side effects were observed including temperature elevation on the first day, transient liver enzyme elevation at high doses and occasional limited hypertension [150]. Considering the results of the bi-shRNA LPX system in EWS and a previous clinical trial in lung cancer (NCT00059605) [151], a phase I active clinical trial was developed that involves pediatric patients (over 8 years old) with advanced EWS (NCT02736565).

**Table 3:** Summary of NPS used in clinical trials (last 5 years) in EWS. Source: ClinicalTrials.gov (accessed on 1 September 2022).

Molecular Mechanism	Interventions	Clinical Trial Identifier	Patients	Phase	Status
Oncogene driver inhibition	Biological: pbi-shRNA™ EWS/FLI1 Type 1 LPX	NCT02736565	EWS	I	Active, not recruiting
DNA damage by topoisomerase inhibition	Onivyde + Talazoparib (Arm A) or Temozolomide (Arm B)	NCT04901702	Recurrent Solid Tumors: EWS; Hepatoblastoma; Neuroblastoma; Osteosarcoma; Rhabdomyosarcoma; Wilms Tumor. Refractory Solid Tumors: EWS; Hepatoblastoma; Malignant Germ Cell Tumor; Malignant Solid Neoplasm; Neuroblastoma; Osteosarcoma; Peripheral Primitive Neuroectodermal Tumor; Rhabdoid Tumor; Rhabdomyosarcoma	I/II	Active, not recruiting
	MM-398 (Irinotecan Sucrosofate Liposome) + cyclophosphamide	NCT02013336	Recurrent or Refractory Solid Tumors: EWS; Rhabdomyosarcoma; Neuroblastoma; Osteosarcoma	I	Recruiting
Depolymerization of microtubules (paclitaxel)	Nab-paclitaxel	NCT03275818	Desmoplastic Small Round Cell, Adult; Desmoplastic Small Round Cell, childhood; EWS; Desmoid	II	Completed
	Nab-paclitaxel	NCT01962103	Neuroblastoma; Rhabdomyosarcoma; EWS; Epitheliod Sarcoma, Soft Tissue Sarcoma, Spindle Cell Melanoma; Melanoma; Osteosarcoma; Histiocytoma; Fibrosarcoma; Dermatofibrosarcoma	I/II	Completed [152]
	Nab-paclitaxel + Gemcitabine	NCT03507491	Cancer	I	Recruiting
	Nab-Paclitaxel + Gemcitabine	NCT02945800	Osteosarcoma; EWS; Rhabdomyosarcoma; Soft Tissue Sarcoma	II	Recruiting
DNA damage by intercalation, disruption of topoisomerase-II and generation of free radicals (doxorubicin)	Disulfiram + Copper Gluconate and Liposomal Doxorubicin	NCT05210374	Relapsed Sarcomas (including EWS)	I	Not yet recruiting
	Liposomal Doxorubicin + MR-HIFU Hyperthermia	NCT02557854	EWS; Rhabdomyosarcoma; Wilms Tumor; Neuroblastoma; Hepatoblastoma; Germ Cell Tumor	I	Withdrawn
	Temsirolimus + liposomal doxorubicin	NCT00949325	Sarcoma (including EWS)	I/II	Completed [153]
	Lyso-thermosensitive liposomal doxorubicin (LTLD) + MR-HIFU Hyperthermia	NCT04791228	EWS; Malignant Epithelial Neoplasm; Rhabdomyosarcoma; Wilms Tumor; Hepatic Tumor; Germ Cell Tumor	II	Not yet recruiting
	Lyso-thermosensitive liposomal doxorubicin + Magnetic resonance high intensity focused ultrasound	NCT02536183	Rhabdomyosarcoma; EWS; Osteosarcoma; Neuroblastoma; Wilms Tumor; Hepatic Tumor; Germ Cell Tumors	I	Recruiting

##### Drug Delivery Systems

Several studies have used NPS to carry anticancer drugs in order to improve drug kinetics and achieve better therapeutic results. On this basis, a small molecule uncharacterized compound ML111 was found to inhibit in vitro the proliferation of six established EWS cell lines with nanomolar potency [154]. Sabei et al., have reported that ML111 encapsulated into a hydrophobic core of PEG-PCL-based polymeric NPS (ML111-NPS) was able to internalize into EWS cell lines and specifically inhibit their viability without altering nonmalignant human cell lines [155]. Moreover, a synergistic effect on the viability of EWS cells resulted from combining ML111-NPS with vincristine in vitro, compared to nonmalignant cells. Moreover, a regression of EWS tumors was observed when using ML111-NPS in vivo, both in monotherapy and in combination with vincristine. No toxicity effects were identified in mice organs with ML111-NPS alone, and with the combination there was a reduction of side effects associated with vincristine [155]. Besides, the use of a hydrolyzed galactomannan (hGM)-based amphiphilic NPS for selective intratumoral accumulation in pediatric sarcoma was also investigated [156]. Coupling of these NPS with the tyrosine kinase inhibitor imatinib could target glucose transporter-1 (GLUT-1), both in rhabdomyosarcoma cells and in EWS PDX with different GLUT-1 expression levels with a 7.5% of efficiency [157], which make them a potential tool against GLUT-1-expressing tumors. Furthermore, Bell et al., employed biomimetic high-density lipoprotein (HDL) NPS. These HDL NPS were able to bind both HDL receptors and scavenger receptor type B-1 (SCARB1), depriving tumor cells HDL and cholesterol, and blocking proliferation in hedgehog-driven EWS cells and medulloblastoma [158].

PARP inhibitors such as talazoparib (TLZ) or olaparib did not show activity in EWS [159,160], although they potentiate the treatment with the DNA alkylating agent temozolomide (TMZ). A nanoformulation of TLZ (NanoTLZ) was reported to be more effective and well tolerated in vivo, while its combination with TMZ elicited an increase in the maximum tolerated dose of TMZ for EWS treatment [159]. Nevertheless, another study showed that the TC71 TLZ-resistant EWS cell line was not affected by frequently administered oral TLZ nor affected by the long-acting PEGylated TLZ [161].

Onivyde (MM-398 or PEP02) is a nanoliposomal formulation of the DNA topoisomerase I inhibitor irinotecan, which is used to treat several solid tumors, although it has a complex pharmacokinetics [162]. Onivyde showed an improvement on the antitumor activity, biodistribution, and a reduction of toxicity in EWS xenografts compared to the current clinical formulation of irinotecan [162]. Currently, a recruiting phase I clinical trial studies the highest dose of MM-398 that can be given safely when combined with cyclophosphamide in patients with recurrent or refractory pediatric solid tumors (NCT02013336). Indeed, an active phase I trial is being conducted with combinations of onivyde with TLZ or TMZ (NCT04901702) to determine the highest tolerable doses of the two combinations (NCT04901702).

A recent work evaluated the albumin-bound (nab)-paclitaxel NPS in PDXs of EWS, rhabdomyosarcoma, and osteosarcoma [163]. These NPS bind to tumor cells that express SPARC, a secreted acidic protein and rich in cysteine that shows a high affinity to bind albumin. Nab-paclitaxel was less bound in SPARC-knocked down (SPARC-KD) compared to SPARC-WT cells [163]. EWS PDX with high expression of SPARC was associated to accumulation of nab-paclitaxel showing better drug responses compared to tumors with lower SPARC levels. Consistently, pediatric tumors that express SPARC were able to accumulate nab-paclitaxel for more extended periods of time [163]. Nab-paclitaxel is being evaluated in several clinical trials including an active phase II clinical trial in monotherapy for patients with EWS and other tumors (NCT03275818). A completed phase I/II multicenter trial (NCT01962103, [152]) showed in EWS patients that the overall response rate was 0%, the disease control rate was 30.8% (4 stable disease), the median PFS was 13.0; and the 1-year OS rate was 48%. The safety of nab-paclitaxel in pediatric patients was confirmed; however, limited activity was observed [164]. Finally, two clinical trials are recruiting patients with pediatric relapsed and refractory solid tumors (NCT03507491); and patients with recurrent/refractory sarcoma (NCT02945800), in which nab-paclitaxel and gemcitabine will be given. However, the results for the EWS arm of NCT02945800 have been published. This clinical trial of nab-paclitaxel and gemcitabine displayed limited activity in a small cohort of EWS patients confirming only one partial response. Moreover, two partial responses after two cycles was observed, but due to the side effects or the progression of the disease, these two patients were withdrawn [165]. The response rate of 9% was similar to other study in EWS patients treated with gemcitabine and docetaxel [165].

Liposomal doxorubicin was designed to increase its therapeutic efficacy while decreasing toxicity. A phase I clinical trial (not yet recruiting) purpose to evaluate disulfiram with copper gluconate and liposomal doxorubicin in treatment-refractory sarcomas (NCT05210374). Another phase I trial is currently running to determine whether delivery of a liposomal doxorubicin called doxil prior to MR-HIFU (magnetic resonance-guided high intensity focused ultrasound) hyperthermia will be safe for the treatment of pediatric and young adult patients with recurrent and refractory solid tumors. Unfortunately, this trial is withdrawn because of the lack of enrollment (NCT02557854). Also-thermosensitive liposomal doxorubicin (LTLD) is the first heat-activated formulation of a liposomal drug carrier to be utilized in human clinical trials. There are two clinical trials with EWS patients. A phase I, recruiting trial that combined LTLD and MR-HIFU in pediatric refractory solid tumors (NCT02536183); and a phase II trial, in which LTLD with MR-HIFU hyperthermia followed by ablation will be studied in subjects with refractory/relapsed solid tumors (NCT04791228).

Finally, complete phase I and II clinical trials showed that combinations of liposomal doxorubicin and temsirolimus were safe and showed efficacy for patients with recurrent sarcoma (NCT00949325 and [153,166]).

#### 3.1.2. Inorganic NPS

Inorganic NPS are metal-based (gold, iron, lead, silver) and metal oxide-based (aluminum oxide, zinc oxide, etc.) particles [146]. Metal-based NPS of gold and silver (Au and Ag NPS, respectively) have been reported to have antitumor effects [156,167,168]; however, Ag NPS can induce general toxicity in non-target organs [169]. These NPS have been also evaluated in the context of EWS at preclinical level. Naumann et al., have developed Au-NPS where selective SN-38 activation in cancer cells is mediated by the EWS specific mRNAs BIRC5 (survivin) and EWSR1-FLI1. In this system, the gold particle is conjugated to the specific mRNA where the complementary SN38-conjugated oligonucleotide anneals. SN38 release will be dependent on the presence of the EWS specific mRNA. The viability of EWS cells treated with SN38-survivin Au-NPS and SN38-EWS/FLI1 Au-NPS was significantly reduced in four EWS cell lines and in murine xenografts [170]. The antitumor activity of silver chloride and silver/silver chloride NPS (AgCl and Ag/AgCl NPS, respectively) has been also investigated in EWS. Treatment of EWS cell lines and a non-tumor cell line with Ag NPS caused a reduction in cell viability specific for tumor cells. Both AgCl and Ag/Ag-NPS increased the percentage of apoptotic cells and ROS production, accompanied with a loss of mitochondrial membrane potential, and lysosomal damage. These effects were specific for tumor cells with minimal effects shown on healthy cells [171].

#### 3.1.3. Carbon-Based Nanomaterials

Carbon-based NPS include fullerenes, carbon nanofibers, diamonds, carbon nanotubes, and graphene. These NPS display multiple properties that make them suitable for drug delivery systems and cancer therapy, as well as imaging, biosensing, or diagnosis [156]. Alhaddad et al., investigated the ability of a siRNA delivery system using diamond NPS [172]. These diamond NPS were coated with a cationic polymer and encapsulated siRNA to inhibit *EWSR1-FLI1*. Because diamond NPS have intrinsic fluorescent properties its internalization into EWS cell lines was efficacious and could be observed directly. Following the internalization, *EWSR1-FLI1* inhibition was observed at mRNA and protein levels in vitro. Finally, cell toxicity was low after treatment with diamond NPS [172].

#### 3.1.4. Hybrid NPS

Hybrid NPS are formed by polymer and organic- or inorganic-based NPS systems that combine the properties of single systems. Consistently, they have lower circulation time and bioavailability, more stability and therapeutic efficacy, being a viable alternative when compared to single systems [173,174].

Hybrid polymerization liposomal NPS (HPLNs) has been developed antibody encapsulated with irinotecan (CD99-HPLN/Ir). Low doses of this hybrid system have shown reduced EWS tumors in xenograft mice and complete tumor ablation, which was more efficacious compared to onivyde and doxil NPS systems. Drug bioavailability was improved six-fold with HPLN and encapsulated irinotecan without CD99 and twelve-fold with CD99-HPLN/Ir in respect to onivyde. Consistently, irinotecan toxic side effects were minimized [173]. Along the same lines, HPLN has been used to deliver siRNA, ASO, or functional CRISPR-Cas9 systems against the fusion oncogene *EWSR1-FLI1*. In vitro experiments resulted in an efficient *EWSR1-FLI1* reduction being the most effective HPLN/CRISPR-Cas9 system. Moreover, CD99-HPLN with CRISPR-Cas9 against encapsulated *EWSR1-FLI1*, reduced EWS tumor growth in vivo [175]. Although promising preclinical results, the potential of these hybrid NPS systems remains to be further evaluated in clinical studies.

## 4. Conclusions and Future Perspectives

The standard therapy for EWS patients based on cytotoxic chemotherapy and radiotherapy has reached a plateau, especially for that subgroup of patients with the worst prognosis. Moreover, patients who survive face debilitating and often life-threatening health consequences as a result of the high toxicity of these therapies. Therefore, and especially considering the young age and potential lifespan of the patients, there is urgent need for finding new therapies to improve the outcome of these patients [176]. Epigenetic-based therapies have changed the targeting focus from extracellular and intracellular signaling to chromatin, where these pathways integrate regulating gene expression in a reversible manner that offers the opportunity for phenotype conversion. The inhibition of epigenetic complexes that regulate the expression and protein stability of the oncogene itself, as well as those cofactors that participate in the modulation of its activity, shows significant achievements in the control of the disease in preclinical studies. Nevertheless, the epigenetic drugs used in clinics have reported modest antitumor efficacy in monotherapy leading to the development of new epigenetic approaches based on the usage of second generation drugs and combinatorial strategies that promote synergistic effects. Further mechanistic approaches should explore differences in drug efficiency between targeting specific enzymatic domains and the effects of depleting the whole protein or inducing its degradation. Immunotherapy, unlike other approaches, induces a therapeutic response not only limited to disrupt a single oncogenic event. Despite promising results of immunotherapy in adult tumors, their application in EWS and other sarcomas has demonstrated poor therapeutic activity due to the immune-cold nature of these tumors. However, different immune strategies are being developed searching for efficient combinations with standard or new targeted therapies, including epigenetic drugs. Besides, other research strategies are focused on the development of more targeted approaches and the reversion of the cold immune landscape of EWS into a hot phenotype. Indeed, our understanding of the crosstalk between the tumor and the tissue microenvironment as well as the basic aspects of the vasculature and hypoxia of EWS would help future direction in immunomodulation therapies [177]. Both for epigenetics and immunotherapy, the introduction of CRISPR screenings to define novel targetable tumor dependencies will postulate promising combinatorial strategies to explore in clinical trials. On the other hand, nanomedicine has evolved to face the lack of specificity, drug resistance and high toxicity rates of both standard regimens and these new therapeutic alternatives. Consistently, NPS as drug delivery systems have reduced both the toxicity associated with cytotoxic drugs and the tolerated dose. This fact raises the possibility to rescue the usage of effective drugs that were discarded in clinics for their side effects by coupling them to novel NPS systems.

Finally, the highly heterogeneity in EWS tumors promote more limitations that also affects the treatment. Firstly, the possibility of developing an appropriate in vivo model, which could contribute to the discrepancy between preclinical and clinical results. However, latest research in patient derived organoid, which recapitulate genetic and phenotypic characteristics of their tissue of origin, support the inclusion of this models in preclinical validation as predictors of response [178]. Secondly, the necessity on finding specific and universally expressed membrane biomarkers that might improve treatment specificity. The discovery of new biomarkers with prognostic value and response to treatment with a more accurate classification of patients, would benefit the creation of a specific treatment plan, also referred to as personalized medicine, that might benefit survival of EWS patients. Taken together, these new therapeutic alternatives and the more effective delivery of drugs by NPS represent a new horizon in treating EWS patients, which is expected to benefit patient survival.

## Figures and Tables

**Table 1:** Summarizing the open clinical trials (last 5 years) targeting epigenetic factors. Source: ClinicalTrials.gov (accessed on 1 September 2022).

Molecular Mechanism	Molecular Target	Drug	Clinical Trial Identifier	Patients	Phase	Status/Ref
DNA methylation	IDH	Ivodesinib	NCT04195555	Advanced Solid Tumors, Lymphoma, or Histiocytic disorders with IDH1 mutations	II	Recruiting
Nucleosome remodeling	LSD1/NURD	Seclidemstat + topotecan and cyclophosphamide	NCT03600649	Ewing Sarcoma (EWS); Myxoid Liposarcoma; Sarcomas with FET-family translocation	I	Recruiting
		Seclidemstat	NCT05266196	EWS; Myxoid Liposarcoma; Desmoplastic Small Round Cell Tumor; Extraskeletal Myxoid Chondrosarcoma; Angiomatoid Fibrous Histiocytoma; Clear Cell Sarcoma; Myoepithelial Tumor; Low Grade Fibromyxoid Sarcoma; Sclerosing Epithelioid Fibrosarcoma	I/II	Enrolling
		INCB059872	NCT03514407	Refractory or relapsed EWS	Ib	Terminated
		INCB059872	NCT02712905	Solid Tumors and Hematologic Malignancy	I/II	Terminated
	SWI/ SNF	Trabectedin + radiation	NCT05131386	Osteosarcoma; Chondrosarcoma; EWS; Rhabdomyosarcoma; Desmoplastic Small Round Cell Tumor	II	Recruiting
		Trabectedin + irinotecan	NCT04067115	EWS	I	Recruiting
		Lurbinectedin with or without irinotecan	NCT05042934	Metastatic and recurrent EWS	I/II	Withdrawn
		Lurbinectedin + irinotecan	NCT02611024	Advanced Solid Tumors; Glioblastoma; Soft Tissue Sarcoma (Excluding GIST) Endometrial Carcinoma; Epithelial Ovarian; Carcinoma; Mesothelioma; Gastroenteropancreatic Neuroendocrine Tumor; SCLC; Gastric Carcinoma; Pancreatic Adenocarcinoma; Colorectal Carcinoma; Neuroendocrine Tumors	I/II	Recruiting
Histone writer	EZH2	Tazemetostat	NCT03213665	Relapsed or refractory: Brain tumors; Solid Tumors; non-Hodgkin Lymphoma; histiocytic disorders with EZH2, SMARCB1, or SMARCA4 gene mutations	II	Active, not recruiting
Histone eraser	HDAC	Vorinostat + chemotherapy	NCT04308330	EWS; Rhabdomyosarcoma; Wilms Tumor; Neuroblastoma; Hepatoblastoma; Germ Cell Tumor	I	Recruiting
Histone reader	BET	BMS-986158 and BMS-986378	NCT03936465	Pediatric Cancer	I	Recruiting

**Table 2:** Summarizing the novel mAb-based therapies in EWS that are in clinical trials. Source: ClinicalTrials.gov (accessed on 1 Septembre 2022).

Molecular Target	Molecular Mechanism	Drug	Clinical Trial Identifier	Patients	Phase	Status/Ref
IGF1R	mAb + targeted therapy	Ganitumab + Palbociclib (targets CDK4 and CDK6)	NCT04129151	EWS; Relapsed EWS	II	Active, not recruiting [120]
	mAb + chemotherapy	Ganitumab + variouschemotherapy regimens (vincristine, vincristine sulfate, ifosfamide, etoposide, etoposide sulphate, doxorubicin, doxorubicin hydrochloride, cyclophosphamide)	NCT02306161	Metastatic EWS; Metastatic Bone Malignant neoplasm; Metastatic malignant lung neoplasm; Metastatic and peripheral PNET	III	Active, not recruiting [119]
GD2	mAb	Hu14.18K322A	NCT02159443	EWS; Melanoma; Neuroblastoma; Osteosarcoma	I	Completed
	ADC	^131^I-3F8	NCT00445965	Brain and CNS tumors; Intraocular melanoma and melanoma; Lung cancer; Metastatic Cancer; Neuroblastoma; Ovarian Cancer; Sarcoma; Small intestine cancer; Retinoblastoma	II	Active not recruiting
AXL	ADC with or without ICI	BA3011 (CAB-AXL-ADC) with or without PD-1 inhibitor	NCT03425279	Sarcomas and refractory sarcomas; EWS; Non small cell lung cancer; Melanoma; Solid Tumor	I/II	Active, recruiting
B7-H3	ADC	^131^I-8H9	NCT00089245	Brain and CNS tumors; Sarcoma; Neuroblastoma	I	Active, recruiting [128]
Endosialin	mAb + chemotherapy	Ontuxizumab (MORAb-004) + gemcitabine and docetaxel	NCT01574716	Metastasic soft tissue sarcomas	II	Completed [129]
PDGFR	mAb + ICI	Olaratumab (LY3012207) + Pembrolizumab (MK3475)	NCT03126591	Soft Tissue Sarcoma	I	Active, not recruiting [127]
	mAb + chemotherapy	Olaratumab (LY3012207) + gemcitabine and docetaxel (ANNOUNCE 2)	NCT02659020	Soft Tissue Sarcoma	I/II	Completed [126]
